# Loricrin: Past, Present, and Future

**DOI:** 10.3390/ijms21072271

**Published:** 2020-03-25

**Authors:** Yosuke Ishitsuka, Dennis R. Roop

**Affiliations:** 1 Department of Dermatology, Faculty of Medicine, University of Tsukuba 1-1-1 Tennodai, Tsukuba, Ibaraki 305-8575, Japan; 2 Department of Dermatology and Charles C. Gates Center for Regenerative Medicine, University of Colorado Anschutz Medical Campus, Aurora, CO 80045, USA; dennis.roop@cuanschutz.edu

**Keywords:** stratum corneum, cornified cell envelopes, loricrin, thiol, ε-(γ-glutamyl) lysine cross-linkage, disulfide cross-linkage, atopy, Langerhans cell

## Abstract

The terminal differentiation of the epidermis is a complex physiological process. During the past few decades, medical genetics has shown that defects in the stratum corneum (SC) permeability barrier cause a myriad of pathological conditions, ranging from common dry skin to lethal ichthyoses. Contrarily, molecular phylogenetics has revealed that amniotes have acquired a specialized form of cytoprotection cornification that provides mechanical resilience to the SC. This superior biochemical property, along with desiccation tolerance, is attributable to the proper formation of the macromolecular protein-lipid complex termed cornified cell envelopes (CE). Cornification largely depends on the peculiar biochemical and biophysical properties of loricrin, which is a major CE component. Despite its quantitative significance, loricrin knockout (LKO) mice have revealed it to be dispensable for the SC permeability barrier. Nevertheless, LKO mice have brought us valuable lessons. It is also becoming evident that absent loricrin affects skin homeostasis more profoundly in many more aspects than previously expected. Through an extensive review of aggregate evidence, we discuss herein the functional significance of the thiol-rich protein loricrin from a biochemical, genetic, pathological, metabolic, or immunological aspect with some theoretical and speculative perspectives.

## 1. Introduction and Overview

### Bricks and Mortar: Which Matters More?

Amniotes have acquired a specialized barrier function in the stratified squamous epithelia to cope with harsh terrestrial conditions [1]. The stratum corneum (SC) not only endows the epidermis with impermeability above the tight junction (TJ) [2] but also serves as mechanical insulation, taking advantage of the durability and resiliency of the cornified cell envelopes (CE) [3]. These structural and functional analogies allow us to compare the SC to bricks and mortar [4]; bricks correspond to corneocytes (terminally differentiated keratinocytes), and mortar denotes lipid bilayers provided from the lamellar granule (LG) secretory system located in the stratum granulosum (SG).

Inborn errors in the formation or function of the SC paracellular barrier can result in autosomal recessive congenital ichthyosis (ARCI), a heterogeneous group of keratinization disorders of various etiologies [5]. For instance, loss-of-function (LOF) in LG lipid transporter ATP binding cassette subfamily A member 12 (*ABCA12*) results in ARCI4B (harlequin ichthyosis, OMIM #2425500), while that in transglutaminase 1 (*TGM1*) impairs corneocyte lipid envelope formation [3] and causes ARCI1 (lamellar ichthyosis (LI), OMIM #242300) [5]. These lines of evidence unequivocally suggest the instrumental roles of the “mortar” (the paracellular lipid) in the maintenance of skin barrier homeostasis, and a universal clinical phenotype is a reactive hyperkeratosis.

In addition, there are the complexity and redundancy regarding the consequence the defects in CE, the “brick”; genetic deletion of either major CE precursors, involucrin (IVL) [6], or loricrin (LOR) [7], do not produce conspicuous phenotypes in mice, and human pathological counterparts have not yet been reported. This may be because the epidermal differentiation complex (EDC) constitutes a diversified family [8,9] and facilitates compensation for the loss of the major CE constituents [7,10,11]. However, as their timely and orderly expression suggests, the CE precursors have distinct functions [3]. This review aims to clarify the functional significance of a major CE protein LOR from a biochemical, pathological, or immunological aspect with some theoretical perspectives.

## 2. Epidermal Differentiation

### 2.1. Cornified Cell Envelopes: Just an Insoluble Matter?

**Figure 1:**
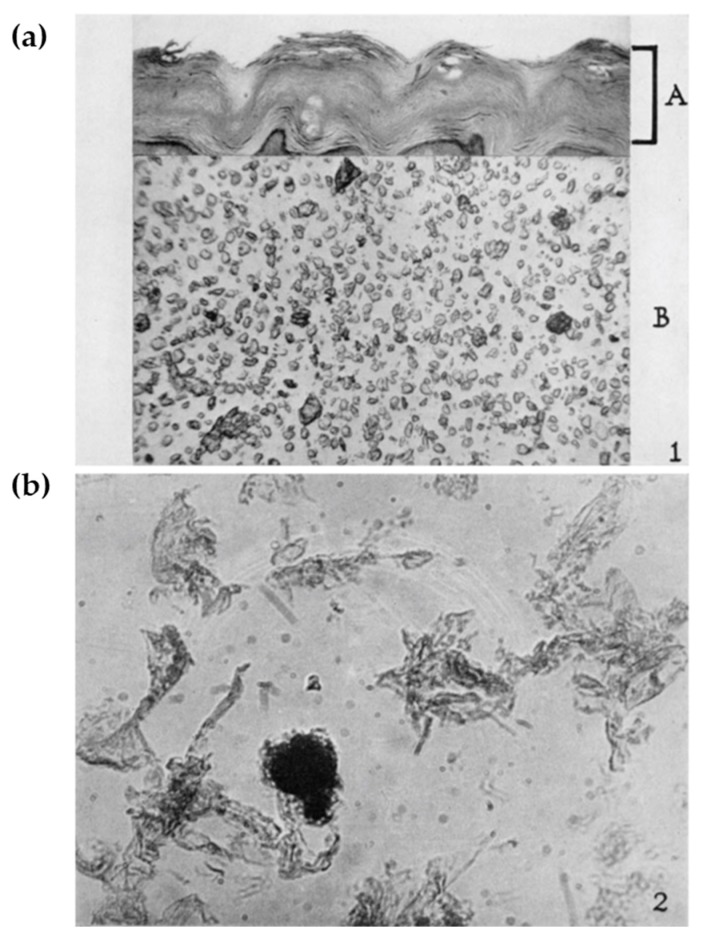
(**a**) A cross-section of human plantar skin showing thickly cornified epithelium (A section). Pulverized callus showing single corneocytes. (**b**) Isolated cell-membrane-like remnants of corneocytes after exhaustive hydrolysis. Adapted from Ref. [13] (Copyright, 1955, by The Rockefeller Institute for Medical Research).

Keratin intermediate filaments (KIF) are the major structural proteins in the epidermis and are indispensable for its barrier function [12]. Perhaps the factor contributing most to the durability of SC is insolubility. CE was initially characterized as a corneocyte cell membrane derived from human callus, which is highly resistant against strong chemicals such as detergent or those with strong alkalinity [13] (Figure 1). Later, it was found that CE insolubility reflects the presence of the ε-(γ-glutamyl) lysine cross-linkage, a covalent cross-linkage that also occurs in the stabilization of collagen, elastin, or blood clotting [14]. TGM catalyzes this reaction that is highly dependent on the extracellular calcium ion concentration [15]. A sequela of reactions ultimately leads to a specialized form of cell death (terminal differentiation) in vitro [16], and the soluble CE precursor IVL was discovered in this experimental setting [15,17].

### 2.2. Loricrin: A Major Cell Envelope (CE) Constituent

Although keratinocytes can be grown and differentiated in submerged cultures [16], purified CE derived from the experimental culture condition is substantially different from that in the physiological state: i.e., human foreskin epidermis [18]. The discovery of the other quantitatively important CE protein LOR brought a solution to this discrepancy [18]. In sharp contrast with the other major CE precursor IVL [15,17], LOR is inherently hydrophobic and insoluble [18]. This peculiar property corresponds with the fact that LOR is easily polymerized via disulfide cross-linkage in the ambient air [18,19], making it suitable as the CE reinforcement protein [3,4].

### 2.3. Tissue Expression of Loricrin: Along with the Air–Liquid Interface

LOR is principally expressed in the SG of mammalian orthokeratinizing epithelia including the epidermis, but not in internal human epithelia [20] (Table 1). This observation again makes a sharp contrast with the expression pattern of IVL that is also expressed in the internal epithelia such as the ano-genito-urinary tracts and medullary thymic epithelial cells (mTECs) [20], all of which mostly face a water–water interface [2]. Collectively, evidence suggests that LOR is the major CE protein required for barrier tissue facing the air–liquid interface, further corroborating the suitability of LOR as the major CE reinforcement protein [3,4].

### 2.4. The Assembly of CE: Building the Brick Wall

**Figure 2:**
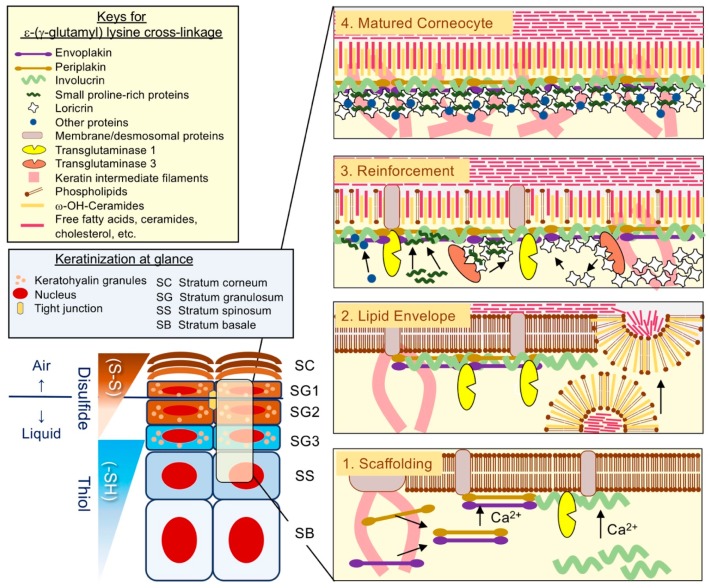
Keratinization at a glance—The epidermis encompasses a gradient of thiol (-SH) to adapt to xenobiotic insults encountered in terrestrial lifestyles. Loricrin (LOR) is a cysteine-rich cornified cell envelope (CE) protein that is critical in establishing the biochemical properties of the epidermis. The three-step model for CE assembly includes: 1. Stage one: scaffolding—Following the microenvironmental rise in Ca^2+^ concentration, involucrin (IVL), envoplakin, and periplakin precipitate at the entire inner surface of the cell membrane. Transglutaminase 1 (TG1) catalyzes ε-(γ-glutamyl) lysine cross-linkages and the monomolecular layer forms. 2. Stage two: lipid envelope—The limited membrane of lamellar granules (LG) delivers long-chain ω-hydroxyceramides. TG1 catalyzes ester cross-linkages between the lipid and IVL. This replaces the cell membrane and forms the corneocyte lipid envelope. Free fatty acids, cholesterol, or ceramides extrude from LGs and form characteristic intercellular lamellae. 3. Stage three: Reinforcement—LOR accumulates in the cytoplasm in the stratum granulosum (SG). Steinert proposed that LOR oligomerization with small proline-rich proteins (SPRRs) (catalyzed by transglutaminase 3) is a critical step to enable an easy translocation to the cell periphery. At the SG1, the only living layer facing an air–liquid interface, LOR undergoes extensive disulfide formation. It thus stabilizes corneocytes after the completion of interchain-intrachain ε-(γ-glutamyl) lysine cross-linkages.

Concurrently with the identification of IVL [15,17], another important epidermal differentiation-related protein, filaggrin (FLG) [21], was identified. This cationic protein accumulates in keratohyalin granules (KG) in the SG, interacts with KIF, and forms the macrofibrils [22]. Based on its biochemical properties, FLG was named after this unique function that enhances KIF aggregation: i.e., filament aggregation [21]. Although a myriad of proteins is incorporated into the CE via ε-(γ-glutamyl) lysine cross-linkage [23,24], understanding the CE assembly process remains essential to comprehend the epidermal differentiation and thus the epidermal barrier function [3,4] (Figure 2).

The earlier phases of cornified CE assembly, which takes place as keratinocytes migrate toward the suprabasal layers, is likely to be essential for the paracellular lipid barrier from a structural perspective. TGM1 catalyzes not only ε-(γ-glutamyl) lysine cross-linkages but also the ester cross-linkage between long-chain ω-hydroxyceramides and IVL [25], as well as the CE scaffold proteins envoplakin (EPL) and periplakin (PPL). This cross-linkage is accompanied with the fusion/extrusion of LG, allowing the formation of the corneocyte lipid envelope in the outer cell layers [3,4] (Figure 2). Indeed, a defective SC paracellular lipid barrier is a characteristic feature of ARCI1 (TGM1 deficiency) [26] or the triple-knockout mice that lack the CE components EPL, PPL, and IVL) [27]. These lines of clinical or experimental evidence suggest the importance of the earlier scaffolding process for the competent lipid-based SC permeability barrier. After completion of the lipid envelope assembly, the keratinocyte cytoskeleton undergoes extensive covalent cross-linking [3,4] and macrofibril formation [22] in the SG. This reinforcement process involves cross-linkages of LOR with KIF, FLG, or small proline-rich proteins (SPRRs) [23]. Morphologically, loss of fluids through the keratinocyte plasma membrane degrades organelles [13] at the air–liquid interface (SG1) [2]. Given the autoxidative property of the thiol, it is highly likely that thiol-rich LOR stabilizes the cytoskeleton via rapid and extensive disulfide cross-linkage formation and promotes corneocyte maturation (cornification) [18,19] (Figure 2).

## 3. Lessons from Mouse Models

Because submerged keratinocyte culture is not a suitable experimental setting to understand the function of LOR for the reasons mentioned above [15,16,18,20], a rodent model is ideal to determine the consequence of the loss or dysfunction of the major CE protein. In particular, if a mouse model recapitulates the human pathology of a known etiology, this would serve as a disease model that brings us a mechanism-based treatment strategy (Table 2).

### 3.1. Vohwinkel Syndrome Transgenic Mouse: Who Done It?

**Figure 3:**
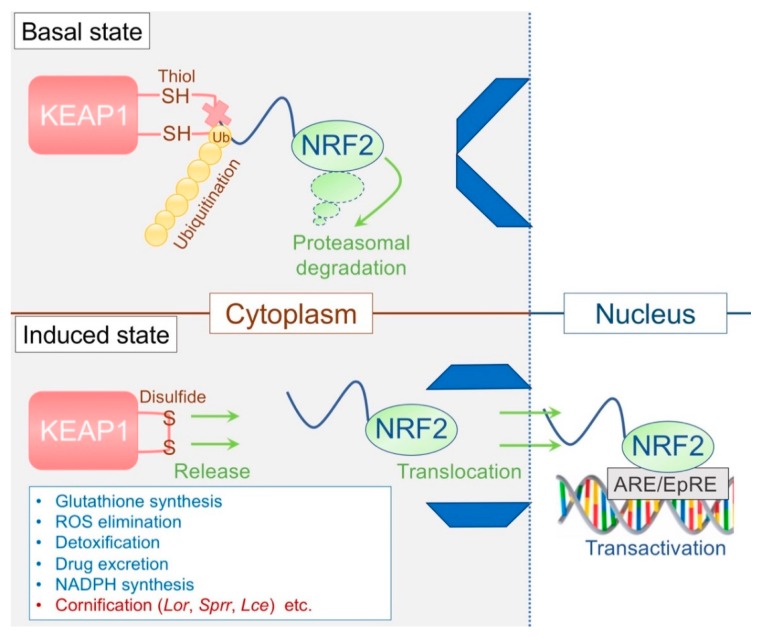
The basics of the Kelch-like erythroid cell-derived protein with cap´n´collar homology-associated protein 1(KEAP1)/ NF-E2-related factor 2 (NRF2) system. KEAP1 acts as a “floodgate” for NRF2 and usually blocks NRF2 entry to the nucleus by facilitating its ubiquitin-mediated degradation. The gate opens upon encountering stresses, and NRF2 translocates into the nucleus and activates a battery of cytoprotective genes. Cornification is an essential aspect of such cytoprotective responses.

Vohwinkel syndrome (VS) with ichthyosis, OMIM #604117, is an ichthyosiform dermatosis caused by dominant-negative mutations in *LOR* [28]. Characteristic features involve palmoplantar hyperkeratosis with small “honeycomb” depressions and progressive formation of pseudoainhum (digital constricting bands) [28] or erythrokeratoderma [29]. VS-related *LOR* mutations result in delayed translation termination, and the C-terminal glycine- or glutamine/lysine-rich domain is replaced with arginine and leucine-rich amino acid sequences. Through morphological observation, it was shown that mutant LOR accumulates in the nucleus [29], while wild-type (WT) LOR moves to the cell periphery and reinforce CE via ε-(γ-glutamyl) lysine cross-linkage [3,30] (Figure 3). However, glutamine/lysine residues of LOR that are used for TGM-mediated ε-(γ-glutamyl) lysine cross-linkages are not supposed to be affected by VS-related mutations in the C-terminus [23]. A mouse model of VS answered this question; extended translation due to the C-terminal mutation generates a nuclear localization signal (NLS), and abnormal nuclear accumulation of LOR hampers the acquisition of the permeability barrier [31]. Moreover, the VS-like phenotype does not require WT LOR, corroborating the dominant-negative effect and autosomal-dominant inheritance observed in the human counterpart [28,31] (Table 2).

### 3.2. Loricrin Knockout Mouse: Dispensable but Indispensable

#### 3.2.1. Dispensability for the Lipid-Based Permeability Barrier

The development of the permeability barrier, the final process of embryonic epidermal stratification, is a highly ordered and patterned process. In mammals, the complete permeability barrier develops from a dorsal-to-ventral aspect and coincides with the CE-reinforcing process in utero [32]. The expression of the major CE reinforcement protein LOR is coordinated with the appearance of LGs. More precisely, LOR accumulates at the cell periphery subsequent to the extrusion of lipid bilayers but prior to profilaggrin processing that yields FLG monomers and natural moisturizing factors (NMFs) [33,34]. This observation unequivocally suggests that LOR is a critical CE reinforcement protein and a vital SC component. However, LOR-knockout (LKO) mice are mostly asymptomatic [7] primarily because the lipid envelope and the LG system develop before LOR expression [33]. This notion is also supported by the spontaneous recovery of the permeability barrier in utero, despite the presence of CE fragility [7]. A series of evidence suggests that LOR is dispensable for the acquisition and function of lipid-based permeability barrier. Although the very mild phenotype of LKO mice was unexpected at that time [35], the meticulous morphological observation [32,33], and the theoretical model of CE assembly [3,4] might have predicted the consequence of the loss of the major CE protein in vivo (Figure 2, Table 2).

#### 3.2.2. Alternative Reinforcement Proteins Small Proline-Rich Proteins (SPRR)/Late Cornified Cell Envelope (LCE)

The EDC comprises the subfamilies of CE reinforcement proteins SPRR and late cornified cell envelope (LCE) that are somewhat structurally related to LOR in terms of amino acid composition or predicted protein structure [11]. In LKO epidermis, SPRR2 and LCE1 are substantially upregulated [7,10,11]. These classes of “alternative” reinforcement proteins are small in molecular weight (around 15 kDa) compared with LOR and are robustly induced during stressed conditions such as ultraviolet B (UVB) irradiation [36,37], wound healing [38], or tape-stripping [39]. In contrast to LOR, the steady-state epidermis expresses smaller amounts of SPRR, but submerged keratinocyte culture or the inner squamous epithelium contains large amounts [40]. Moreover, SPRR does not appear to be merely a reinforcement CE constituent; it has ubiquitous ε-(γ-glutamyl) lysine cross-linkage partners including CE scaffold proteins (EPL/IVL) or KIFs (K5/K6) [41]. By contrast, LOR is preferentially engaged in cross-linkages between differentiated keratins (K1/10, K2) or FLG [23]. From a functional perspective, SPRR provides versatile anti-inflammatory or anti-oxidative properties [40,42] and accommodates specific physical requirements or functions in different tissues [41]. Therefore, abundant expression of SPRR2s or LCE1s in LKO mice [10,11] appears to compensate abrogated biomechanical properties required for the epidermal barrier function, rather than the permeability that is maintained by the LG system. Additionally, from a phylogenic perspective, it is conceivable that the diversified EDC subfamilies [1,8] have evolved to accommodate various xenobiotic assaults in the terrestrial life mentioned above.

#### 3.2.3. Indispensability for Cornification

The ε-(γ-glutamyl) lysine cross-linkage is a powerful covalent bond that maintains tissue integrity in the epidermis, and inborn errors of this cross-linkage result in pathological conditions. TGM1 deficiency impairs CE formation (ARCI1, Table 2), TGM5 deficiency causes premature desquamation (peeling skin syndrome 2 (OMIM # 609796)), and TGM3 deficiency impairs hair shaft formation (uncombable hair syndrome 2 (OMIM # 617251)) [43]. CE is an extremely insoluble macromolecular structure composed of lipid and proteins bonded together via TGM-mediated ε-(γ-glutamyl) lysine cross-linkages [3,4,13,15] (Figure 1). For instance, LOR is an important TGM substrate that undergoes differential post-translational modification, according to TGM isozymes; TGM1 mediates LOR polymerization, and TGM3 induces its conformational changes [44] (Figure 2). However, performing a detailed analysis of CE composition is far from simple. Specifically, covalent bonds among CE components mask specific epitopes for immunodetection, obscuring a proper view of the structure in situ. Steinert and colleagues performed a series of ingenious studies using foreskin epidermis and deduced a model for the CE structure [3] (Figure 2). At that time, isolated CE was digested with proteinase K, obtained peptides were fractionated by high-performance liquid chromatography (HPLC), and peptide species containing glutamine/lysine residues were subjected to Edman degradation [23]. Through this elaborate approach, they found that LOR cross-linkage partners involve SPRR, K1/K10, FLG, and elafin, as well as LOR itself [23]. Because submerged keratinocyte culture yields minimal LOR expression levels, as mentioned above [16], LKO mice would serve as an excellent model to analyze immature CE phenotypes in vivo. We isolated LKO CE, lysed it with trypsin, and performed shotgun proteomic analysis [45] instead of HPLC, as Steinert and colleagues did [23]. The most notable difference we found was the amount of FLG (monomer or multimers); LKO CE contained a remarkably decreased amount of FLG monomers compared with WT CE and differentiated KIF K1/K10 ([46] and unpublished data). This observation is in line with the Steinert’s structure model [3,23] and supports the notion that LOR fulfills an adapter-like function that organizes substrates for ε-(γ-glutamyl) lysine cross-linkages in the CE [11] (Figure 2).

After the completion of TGM-mediated cross-linkage in the SG, LOR enhances intrachain-interchain disulfide cross-linkage upon exposure to the ambient air in the SG1, the only living epidermal layer facing an air–liquid interface [2] (Figure 2). This conformational change provides the corneocyte with elasticity and resiliency as well as mechanical stability [18,19]. Indeed, we found that LKO mice were markedly susceptible to UVB irradiation despite the abundance of unlinked FLG monomers and the photo-absorbing FLG-breakdown products, urocanic acids (UCAs) [47]. Moreover, LKO mice exhibited increased NMF in SC and LG biogenesis ([47] and unpublished data), the latter of which is presumably associated with the relative abundance of the lipid-envelope-forming CE scaffold proteins EPL, PPL, and IVL ([46] and unpublished data). Thus, evidence suggests that LOR is an indispensable thiol-rich EDC protein that stabilizes differentiated keratinocytes via covalent ε-(γ-glutamyl) lysine/disulfide cross-linkages [18,19,23,46,47].

### 3.3. The KEAP1/NRF2 System: The Epidermal Keeper and Striker

#### 3.3.1. The Epidermal Thiol Gradient and Cornification

The Kelch-like erythroid cell-derived protein with cap´n´collar homology-associated protein 1 (KEAP1)/NF-E2-related factor 2 (NRF2) system constitutes important cytoprotective machinery and has recognized as a primary defense mechanism against environmental insults [48]. KEAP1, a thiol-rich protein, senses electrophiles, and NRF2 induces the coordinated activation of cytoprotective genes that are related to phase II detoxification [48]. KEAP1 subjects NRF2 to ubiquitin-mediated proteasomal degradation in the cytosol and thus represses NRF2 in the steady state [48,49,50,51] (Figure 3). Accordingly, constitutive activation of the transcription factor NRF2 can be obtained through suppressing KEAP1 [48]. Although *Keap1*-deficient mice exhibited enhanced expression levels of phase II detoxification enzymes, the phenotype was unexpected and remarkable; the mice died of malnutrition caused by hyperkeratosis in the esophagus/forestomach with concomitant overexpression of LOR located downstream of NRF2 [52]. This lethal phenotype was rescued entirely in *Keap1*/*Nrf2* double-deficient mice [52]. Notably, the skin phenotype of *Keap1*-deficient mice was also orthohyperkeratotic and somewhat resembled ARCI, in which enhanced TGM activity and LOR expression are commonly observed [53,54]. Later, K5 promoter-driven expression of the constitutive active *Nrf2* mutant reproduced a skin phenotype similar to *Keap1*-deficient mice [55]. Moreover, a meticulous transgenic approach [56] revealed that NRF2 establishes an inherent epidermal thiol gradient and protects against UVB [56,57]. These lines of evidence suggest that the KEAP1/NRF2 system is a critical regulator of cornification [58], the ultimate form of cytoprotection in the epidermis (Figure 3, Table 3).

#### 3.3.2. Disrupted Epidermal Thiol Gradient and Recovery

LOR was identified initially as the thiol-rich protein [18] that constitutes the sulfur-rich KG L-granule [30] and promotes ε-(γ-glutamyl) lysine cross-linkage of the cytoskeleton in the SG [3,18,19,23,46]. At the air–liquid interface SG1 [2], the NRF2-downstream target LOR stabilizes the corneocyte through disulfide cross-linkage [46,47]. Because thiols are redox-sensitive, they serve as an antioxidant but are easily subjected to autoxidation in the ambient air. The redox-driven KEAP1/NRF2 system maintains the epidermis-intrinsic thiol gradient and promotes cornification along with its downstream target *Lor* [52,56]. This autoxidation machinery looks like an entirely reasonable way to utilize the inherent instability of thiols for epidermal cytoprotection against innumerable xenobiotic stimuli. Now we knew that LOR is indispensable for cornification but did not understand how the epidermal biological system responds to truncated epidermal terminal differentiation. LKO mice provided answers to this question.

#### 3.3.3. Dominant-Negative NF-E2-related factor 2 (Nrf2) Mice

The transcription factor NRF2 has a *cis*-regulatory element termed the antioxidant responsive element (ARE) or electrophile responsive element (EpRE) (Figure 3). This consensus sequence (TGA^G^/_C_NNNGC) is found in NRF2-downstream target genes [48]. The unexpected phenotype of *Keap1*-deficient mice has made us recognize that EDC genes are under the control of the KEAP1/NRF2 system [52]. *Sprr2*s are upregulated to compensate for the loss of LOR in the epidermis [7]. They reside in close proximity to the *Lor* locus in the EDC [11] and harbor AREs in the promoter region [10,11] analogously to that observed in phase II detoxifying genes or *Lor*. Based on this, a dominant-negative experimental approach was undertaken [10]. This transgenic mouse expresses a mutant *Nrf2* (Δ*Nrf2*) under the control of the *Lor* promoter [10]. Introducing the transgene into LKO mice abrogated the compensatory response in utero and led to the retarded acquisition of the permeability barrier [10] (Table 3). Through use of an in vitro colorimetric reporter assay [59], it was concluded that the increased electrophilic potential of amniotic fluid during the late gestational phases (embryonic day (E)14.5 vs. E16.5) serves as an environmental cue to stimulate the KEAP1/NRF2 system in the SG1 [10] (Table 2). However, it should be noted that *Nrf2*-deficient LKO (double-knockout) mice do not exhibit such a lethal barrier defect (unpublished observation). This discrepancy may be due to the presence of other *Nrf* family members that putatively compensate for the loss of *Nrf2* [60]. Alternatively, the activation of NRF2 is explicitly required for the developmental process of the SC, analogously to the retinoid receptor signaling [61,62,63].

## 4. Epidermal Microenvironment and Immune Homeostasis

The epidermis is composed of mostly keratinocytes. However, there is ample evidence that resident leukocytes, which constitute relatively small populations, play a pivotal role in the regulation of tissue homeostasis. For instance, the dendritic epidermal T cell mediates a rapid tissue-repairing response by directly recognizing a stress-induced keratinocyte self-antigen [64] following wounding [65] or carcinogen exposure [66], and it constitutes an integral part of epidermal stress surveillance [67]. In addition, the Langerhans cell (LC), a canonical epidermal resident antigen-presenting cell, regulates the adaptive immune system through regulatory T cell (Treg) memory [68,69]. Recently, it was shown that this dominant mode of tolerance in the skin facilitates efficient tissue-repairing responses such as hair regeneration [70], possibly through the prevention of devastating immune responses against daily encountered antigens such as those from commensal microorganisms [71,72] and self-antigens [69]. Given that LOR plays the aforementioned pivotal roles in epidermal terminal differentiation, the question remains of what LOR contributes to epidermal homeostasis from an immunological perspective. Furthermore, what kinds of cross-talking machinery between keratinocytes and leukocytes [73] occur behind these “quasi-normal” [74] phenotypes in LKO mice?

### 4.1. Lessons from Filaggrin-Deficiency: Flaky or Leaky

Atopic eczema, or atopic dermatitis (AD) [75], is a common inflammatory skin disorder and a chronic form of allergic contact dermatitis (ACD). In 2006, McLean and colleagues discovered that LOF variants in *FLG* result in ichthyosis vulgaris (a common dry skin, Table 2) [76] and are a major predisposing factor for AD [77]. AD in childhood is the very first condition that precedes sequelae of allergic manifestations (e.g., asthma, food allergy, allergic rhinitis, etc.) [78]. A strong association between AD (with *FLG* mutations) and extracutaneous allergic phenotypes was later found [79,80,81]. However, AD is an oligogenic and multifactorial disorder that is not merely associated with a defective skin barrier [82]. Why then does the *FLG*-locus (ATOD2 (OMIM #605803)) have by far the most robust relationship among EDC genes? From an evolutionary perspective, *FLG*-null alleles are considered to have been advantageous during pandemics [83], possibly through enhancing SC “leakiness” and antigen uptake above the TG [84]. If so, even though percutaneous sensitization preferentially generates a local and systemic type 2 immunological memory, i.e., atopy [85], how can extracutaneous allergic symptoms be managed with percutaneous immunotherapy [86,87,88]?

Aberrant activation of epidermal innate immunity caused by interleukin-1 (IL-1) family signaling causes various inflammatory skin disorders. Pustular psoriasis (PSORS14 (OMIM #614204)) is caused by uncontrolled IL-36 signaling [89,90], and forced epidermal expression of IL-33 causes an AD-like skin phenotype in mice [91]. Similarly, it is also becoming evident that immune activation in the epidermis does not necessarily depend on the presence of microorganisms on the skin surface [92,93,94]. In short, the immune microenvironment in the epidermis [95] controls innate and adaptive immunity [96] and thus profoundly affects the outcome of the immune response: i.e., the immune effector function [97]. In this respect, the constant cross-talk between keratinocytes and leukocytes [73,90] may prevail over any external assaults [92,93,94]. In other words, the tissue status [97] may matter more than mere “leakiness” of the epidermis in immune system activation.

### 4.2. The F-Granule and L-Granule: “La Raison D’êTre” of the Epidermis

**Figure 4:**
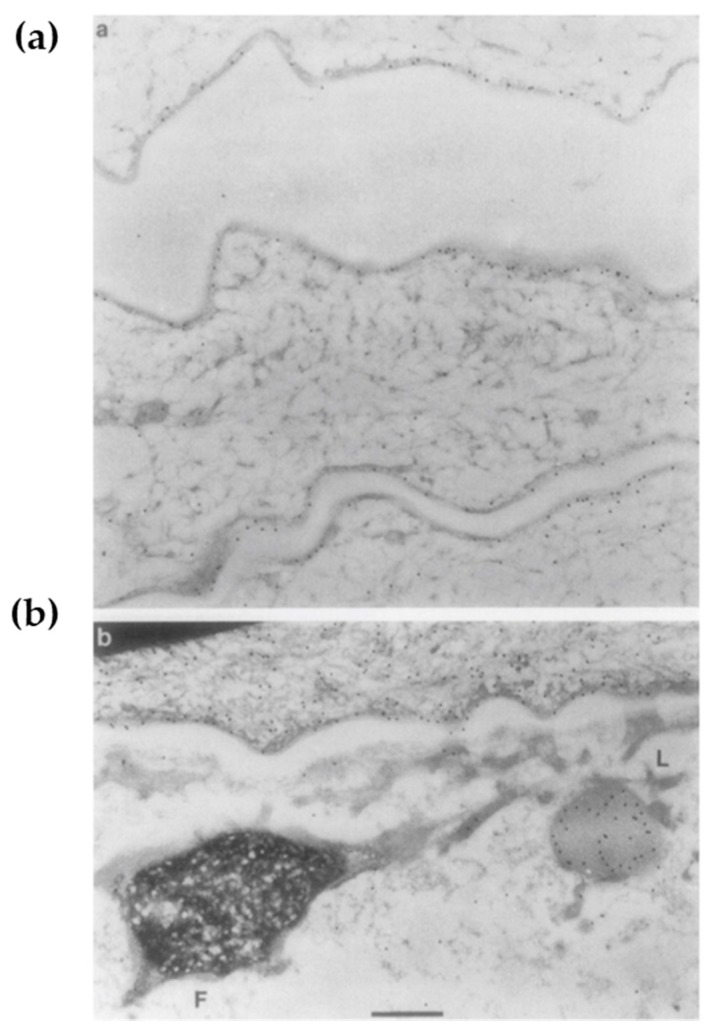
The F-granule and L-granule: “la raison d’être” of the epidermis (adapted from Ref. [30]). Immunoelectron microscopic observation of newborn mouse epidermis labeled with anti-loricrin antibody and protein A-gold (15 nm). In the stratum granulosum (SG), filaggrin-containing F-granules and loricrin-containing L-granules are visible. (**a**) Labeling is predominantly confined to the cell envelope (the cell periphery) at the outer stratum corneum. (**b**) Strong labeling of an L-granule in the SG. In the innermost corneocyte, there is labeling of envelope and cytoplasm. Bar = 0.5 µm.

After the formation of the lipid envelope and LG biogenesis, keratinocytes undergo cornification [98]. This biological process involves ε-(γ-glutamyl) lysine cross-linkages among cytoskeletal proteins K1/10, FLG, and LOR, followed by the somewhat abrupt disappearance of cell organelles [99] in the SG1 [2]. In this process, the interaction between the profilaggrin-containing F-granule and the LOR-containing L-granule along with KIF is critical [30]. Upon transition from SG1 to SC, the L-granule moves to the cell periphery, while the F-granule remains in the cytoplasm [30]. This classic ultrastructural observation (Figure 4) may reflect the essential step in epidermal terminal differentiation. Although NLS-harboring VS-related mutant LOR does not move to the periphery and disrupt the permeability barrier [23], the L-granule itself is dispensable for the permeability barrier [7] but mandatory for cornification [7,46,47]. Likewise, F-granule abnormalities reflect aberrant cornification rather than a compromised permeability barrier [98]. Profilaggrin undergoes proteolytic processing mediated by many peptidases including caspase-14 (CASP14) [33,34]. Reflecting the absence of its enzymatic activity, *Casp14*-deficient mice have a decreased amount of NMFs/UCAs in the SC and exhibit increased susceptibility to UVB [98,100] (Table 2). However, given that photons in UVB radiation transfer energy and directly cause structural damage, cytoskeletal integrity may have a more profound impact on photo-protection than do the photo-absorptive small molecules [47]. *Casp14*-deficient F-granules exhibit a swollen, mottled phenotype that is reminiscent of *Tgm1*-deficiency [101] (Table 2), suggesting that CASP14 not only degrades FLG [98] but also promotes the cytoskeletal cross-linkage in the SG [101]. Similarly, UVB-susceptible LKO corneocytes are abundant in unlinked K1/K10, FLG, and CASP14 [46,47]. These lines of evidence suggest that CASP14 also plays an essential role in cornification, which essentially is a specialized form of cell death [102], leading to cytoprotection.

Mouse models have thus far elucidated the functions of major molecules that mediate cytoskeletal cross-linkage in corneocytes such as KIFs [12,103,104], IVL/PPL/EPL [27], FLG [105], LOR [7,10,11,46,47], and TGM1 [101] (Table 2). Notably, the absence of KG proteins FLG [105], LOR [7], or even scaffolding proteins [27] or KIFs [12] does not affect LG biogenesis. By contrast, defects in the formation of the lipid envelope [27,101] or LG-mediated lipid transport [106,107] affect water retention. Intriguingly, the CE scaffold [14,15] and the LG system are also present in non-keratinizing inner squamous epithelia such as the esophagus [108], buccal mucosa, or the vagina [109]. However, the expression of FLG and LOR is confined to the epidermis with the exception of the keratinizing hard plate mucosa [20,110] (Table 1), suggesting that these major KG proteins are instrumental in covering the dry, outer squamous epithelium. By analogy, recent phylogenetic evidence may give us a clue to understanding its significance; cetaceans such as whales or dolphins have lost the *FLG* gene to adapt their skin to aquatic lifestyles [1,111,112].

### 4.3. Atopy: Imprinted Cutaneous Immunological Memory?

The percutaneous entry of foreign antigens preferentially generates a type 2 immunological memory and atopy, resulting in the sequelae of extracutaneous allergic symptoms sometimes called the “atopic march” [85,113]. By contrast, the oral route, which is a more physiological way of encountering the “non-self”, results in Treg memory [85,114]. However, this is not a fundamental principle. Hay fever [88] or food allergy [86] can be managed with percutaneous immunotherapy. The topical application of a dinitrophenol hapten dinitrothiocyanobenzene (DNTB) induces Treg-mediated tolerance (Treg memory) [68] and attenuates ACD against a cross-reactive hapten 2,4-dinitrofluorobenzene (DNFB) [115] just as efficiently as orally administered DNFB [114]. In this respect, Pickard et al. presented suggestive data that an epidermis-intrinsic biochemical property may be responsible for cutaneous immunological memory [116]. They found that compared with the “tolerogenic” DNTB, “allergic” 2,4-dinitrochlorobenzene (DNCB) depletes free thiols in the epidermis extensively, suggesting the importance of the thiol-rich layer that reacts with haptens in the SC (or SG1) [116]. As noted above, the KEAP1/NRF2 system not only establishes the epidermal gradient of the tripeptide glutathione (GSH; a source of free thiol) but also promotes cornification by transactivating *Lor* in the SG [10,52]. Indeed, *Nrf2*-deficient mice can exhibit an impaired ACD response against haptens [117]. In mammalian squamous epithelia, the epidermis has abundant LOR expression, but oral and esophagus mucosa has little, suggesting that LOR preferentially covers the dry, external epithelium and serves literally as an armor for terrestrial life [18,20]. Despite the presence of epithelium-resident LCs [108,118], the oral route preferentially generates Treg memory, while the percutaneous route favors a type 2 immunological memory [85]. Accordingly, the relative immune privilege of the inner squamous epithelia, in comparison with the epidermis, appears to facilitate the induction of tolerance (Treg memory) via the oral route [119]. This evidence suggests that anatomical properties of the epithelium determine the classes of immunological memory.

### 4.4. Immunoanatomy of the Epithelium: It Is Not What It Is Made Of, but the Reaction on the Surface That Matters

When a simple comparison is made, the primary structural difference between the simple columnar intestinal epithelium and the stratified squamous epithelium may be the presence of the LG-mediated, lipid-based permeability barrier [109,120]. Given that the TG-based permeability barrier is present in both types of epithelia [6,108,121], this crucial structural difference may account for the gut microbiota having more profound effects on the regulation of the peripheral immune system compared with skin microbiota [122,123,124,125]. Similarly, conversation between the gut microbiota and the host is required for both physiological [126] and pathological regulation of the systemic immune system [122,123,124]. The classic example is the spontaneous chronic enterocolitis in IL-2- or IL-10-deficient mice [127,128] requiring resident enteric bacteria [127,129], as do the T-cell receptor αβ-deficient mice [130,131]. Although the skin also develops a Treg memory against commensals [71,73], compared with the gut, the bacterial load on the skin surface itself does not greatly affect the gross immune effector function [73,92], even in the context of a defective epidermal barrier [93,94]. Instead, virulent clones appear to emerge by escaping from the activated innate immune system [132,133,134] as in positive Darwinian selection [135]. This dysbiotic microbiota feast on the inflammatory spoils [136], resulting in a vicious cycle between AD and *Staphylococcus aureus* colonization [132].

**Figure 5:**
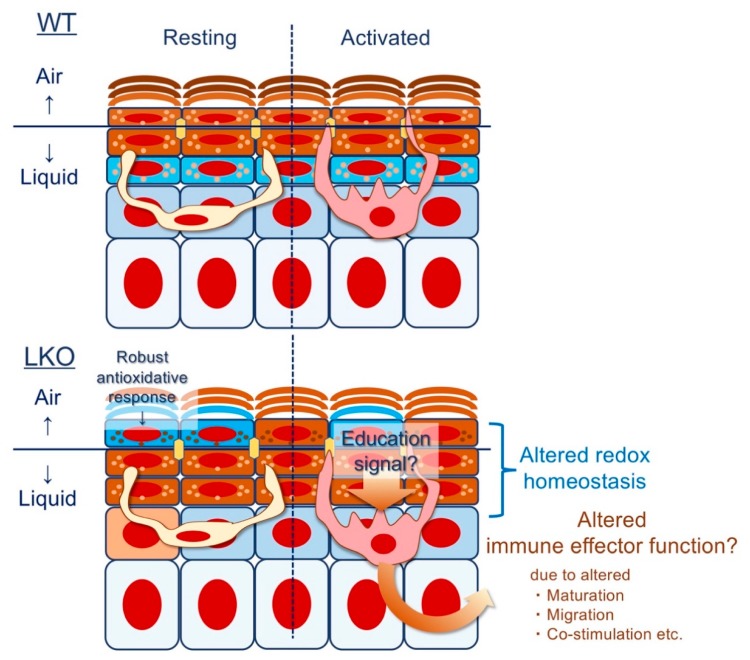
Epithelial imprinting of immunological memory: does LOR instruct the Langerhans cell (LC)? Epidermal Langerhans cells (LC) express tight junction proteins, project dendrites, and sample self-antigens and foreign antigens in the steady state. Loricrin knockout (LKO) epidermis exhibits a robust anti-oxidative response upon exposure to the ambient air, and keratinocyte antioxidants such as small proline-rich proteins (SPRR) or late cornified cell envelope (LCE) are upregulated. However, both SPRR and LCE cannot fully compensate for the biochemical property of loricrin (higher-order structure that enable a prompt intra- or inter-molecular disulfide formation); therefore, redox homeostasis (or metabolic environment) would be profoundly affected in the absence of loricrin. The altered epidermal microenvironment could condition LC (or other dendritic cell subsets) to limit an excessive inflammatory response.

Through the immunoanatomic analogy between the gut epithelium and the epidermis [137], Kubo et al. visualized that activated LCs project dendrites above the TJ to sample external antigens [84] (Figure 5). From a functional perspective, LCs constantly uptake epidermal self- and non-self-antigens [84,138,139] and emigrate to the draining lymph nodes [139] more slowly than blood-supply-derived dermal dendritic cells (DCs) do [139,140]. This steady-state antigen transport from non-lymphoid tissue elicits tolerance against self-antigens by imprinting a Treg memory [141]. Accordingly, the LC, which can be described as a “macrophage in a DC’s clothing” [142], protects from the potentially harmful cytotoxic immune response of ACD [68,143,144]. These lines of evidence suggest that in a similar way as intestinal DCs, cutaneous DCs preferentially induce immunological tolerance (Treg memory). From an immunoanatomical perspective, what lessons can we learn by comparing the epidermis and mucosal squamous epithelia that rarely express FLG/LOR (in the hard plate mucosa) [20,110]?

Of course, the significant difference is the presence or absence of the SC, which provides a far more effective impermeability [62] compared with inner squamous epithelia. The development of the SC permeability barrier takes place on E16.5 from the dorsal-to-ventral aspects like a wave [32]. This final process of epidermal differentiation (keratinization), which leads to the abrupt specialized cell death in the SG1 [16], involves many biological changes such as rapid influx of intracellular calcium ion [145], disappearance of the TJ permeability barrier [146], or the degradation of organelles [13]. However, based on the structural “bricks and mortar” analogy [4], acquisition of the SC permeability barrier depends on the redox-based prompt cytoskeletal cross-linking (bricks) [10,27,147] and timely attachment of extruded LG content (mortar), resulting in an ordered lamellar structure [33,61]. Now that we know that LOR on the “brick” side is dispensable for SC permeability barrier [7], we next focus on endogenous metabolic signaling with a fresh perspective.

### 4.5. Metabolic Regulation of the Epidermal Barrier: The Epidermal “A and D”

The retinoic acid (RA) signaling pathway is a critical regulator of epidermal differentiation [61]. Using the dominant-negative approach, Imakado et al. have elegantly shown the primal importance of RA receptor (RAR) signaling in the development of the SC permeability barrier [61]. In this mouse model, the mutant RAR-α (*Rara*) is expressed exclusively in the differentiated epidermis (Table 4). Abrogated RAR signaling in differentiated keratinocyte results in desiccation [61]. In contrast to the deficiencies of TGM1 [26] or ABCA12 [106] (Table 2), blockade of RAR signaling does not cause LG abnormalities but specifically abrogates the formation of SC lipid bilayers [61]. This “cracked mortar” similarly induces premature cornification as observed during vitamin A deficiency (VAD) [62]. This explains why vitamin A analogs are useful for managing skin conditions such as ARCI or acne caused by uncontrolled cornification in the interfollicular epidermis (ARCI) [148] or the follicular infundibulum (acne) [149], respectively. Likewise, VAD leads to ectopic cornification of mucosal squamous epithelium or transitional epithelium [150].

By contrast, excessive RA suppresses cornification and induces mucous metaplasia in cultured embryonic skin [151] or psoriatic epidermis [152]. From a biochemical perspective, RA reduces the expression levels of *Lor* and induces those of *Ivl* in keratinocytes grown either in submerged cultures [153] or at the air–liquid interface [154,155]. This peculiar property facilitates the structural requirement of each CE component. For the development of a competent SC permeability barrier that depends on the formation of intercellular lipid bilayers, the earlier scaffolding CE components EPL/PPL/IVL are mandatory [27], but the reinforcing component LOR is dispensable [7] (Table 2).

Although VAD often accompanies ectopic cornification of the mucosal squamous epithelium or the transitional epithelium [150], vitamin A (retinol) is known as a critical regulator of the immune response. For instance, VAD is associated with pathological conditions in the gut such as persistent diarrhea and infection [156], and oral vitamin A supplementation ameliorates these symptoms by inducing gut-homing T cells, particularly Tregs [157,158]. It is considered that direct dietary intake through the digestive tract or the bile derived from the storage in the liver is the source of lipophilic nutrient vitamin A [157,158]. In the small intestine (SI), lamina propria DCs that are positive for adhesion molecule CD103 efficiently transmit environmental cues to the draining mesenteric lymph nodes and regulate the immune effector function [156,157,158]. The SI epithelium is capable of producing RA locally through metabolizing dietary vitamin A present in the intestinal lumen [157,158], and epidermal keratinocytes can metabolize vitamin A [159] or RA [160]. However, because living epidermal layers are highly insulated from the outside, the tissue fluid in the dermis is the source of this nutrient [161,162].

Unlike the gut, the skin requires another essential metabolic cue for its competent immune effector function: vitamin D [163]. The active form of the vitamin D metabolite calcitriol (1α,25-(OH)_2_D_3_) activates vitamin D receptor (VDR) signaling and efficiently induces skin tropism [164]. This tissue-specific tropism is achieved through the regulation of CC chemokine receptors in naive T cells; calcitriol upregulates CCR10 that promotes skin tropism but downregulates CCR9 that promotes gut tropism [163].

For efficient cellular signal transduction, both RAR and VDR signaling pathways require heterodimer partner retinoid X receptors (RXRs). Acquired ablation of RXRs [165] presumably causes a relative abundance of epidermal VDR signaling and results in an AD-like phenotype [166,167]. Analogously, topical application of calcipotriol (a low-calceminc calcitriol analog) mirrors the AD-like phenotype [167]. Conversely, orally administered 9-*cis*-RA (alitretinoin), an RXR agonist, is useful for the management of chronic hand eczema [168]. Together, these data suggest that RAR signaling predominates over VDR signaling in steady-state epidermis. Moreover, because AD (or eczema) is closely associated with dry, scaly skin [27,77], the functional dichotomy between the RAR and VDR signaling pathways may also be applied to epidermal terminal differentiation. RAR signaling is essential in the maintenance of the SC paracellular barrier (mortar) rather than cornification (brick) [61,62]. By contrast, as adult VDR-deficient mice exhibited impaired assembly of KG/KIF in the SG [169], VDR signaling is essential in the maintenance of cornification. Given this, we speculate that these two signaling pathways synergistically maintain the epidermal barrier function, and an unbalanced antagonism may lead to dry, flaky skin that can lead to the development of eczema (or an AD) [166,167]. Alternatively, the vitamin A or vitamin D metabolism-based antagonistic dichotomy might simply reflect the specific immune effector function optimized for the outer body wall [97,163]. Finally, because both the VAR [153,154,155] and VDR [169] pathways affect *Lor* expression, it may be intriguing to analyze the consequence of altered metabolic regulation in the SG, possibly caused by absent LOR.

### 4.6. Epithelial Imprinting of Immunological Memory: Does Lor Instruct the Langerhans Cell (LC)?

Given that AD is interpreted as a chronic form of ACD [78], we return to the evidence presented by Pickard et al. [116]; the development of ACD likely depends on the thiol-band that appears abruptly at the SG1 and gradually disappears at the upper SC [2,147]. Indeed, LKO mice, which lack the redox exchange in the SG1 to SC (even in the presence of massively upregulated alternative CE constituents [7,10,11] and unpublished data), are susceptible against common environmental mutagens such as UVB [47] or aromatic hydrocarbons [170]. Unexpectedly, and perhaps more importantly, LKO mice have a markedly attenuated ACD response [170], suggesting that neoantigen-specific cell-mediated cytotoxic immune responses [171] are attenuated. From a clinical perspective, this makes sense because AD patients [172] sometimes experience widespread viral/bacterial infections [173], reflecting ineffective cutaneous immunosurveillance against intracellular pathogens. In this light, LOR deficiency itself may induce an immune-privileged state. Fas (CD95)/Fas ligand (FasL; CD95L)-mediated apoptosis of infiltrating lymphocytes can be tissue-protective and is essential for immune privilege in the eye [174] or the testes [175]. However, in the epidermis, severe genotoxic damage such as UVB irradiation activates the Fas/FasL pathway [176], refuting its active engagement in the steady-state LKO epidermis [47]. Alternatively, given that LOR expression in humans is confined to the epidermis (the hard plate mucosa is an exception but lacks the SC) [20], it is tempting to speculate that the peripheral immune system directly recognizes truncated epidermal differentiation and optimizes an immune effector function [97] suitable for the recovery of the epidermal barrier [7] (Figure 5).

In response to microbes or external injury, polymorphonuclear neutrophils (PMNs) are promptly recruited to the skin surface (more precisely, the SG), resulting in the psoriasiform tissue reaction [177,178]. Although the tissue response is instrumental in the rapid eradication of non-intracellular microorganisms, aberrant activation of PMNs results in tissue-destructive consequences [179], and impaired corneocyte maturation (parakeratosis) is the epitome of such inflammatory destruction [177,178]. If the inflammatory response is in a chronic phase, the secondary lymphoid organ is enriched with immature LCs, probably as an attempt to contain excessive tissue responses [180]. Given that most forms of inflammation are self-limited [181], LKO mice, which exhibit a robust recovery of the epidermal barrier [7,10,11], suggest a critical aspect of skin homeostasis regulation during the resolution phase of cutaneous inflammation. Including LCs, DCs are conveniently poised at the interface of the barrier tissue and comprise an integral part of the permeability barrier beneath the SC [2,84] through regulation of TJ protein expression [137]. Therefore, it would be reasonable to hypothesize that LCs “sense” epidermal metabolic or biochemical cues and tailor the resultant immune effector function (Figure 5). Because thiol oxidation on the DC cell surface triggers endocytosis and suppresses maturation [182,183,184], it could be speculated that the absence of thiol-rich L-granule directly affects LC phenotypes in situ. From a broader perspective, this could be a concise explanation for why percutaneous antigen exposure results in atopy, while permucosal routes [79,85,114] or mTECs [20,185] induce tolerance. In other words, the presence or absence of the thiol-band at SG1 [147] could be a tissue-derived instructive factor of the squamous epithelium, determining immune effector function [116]. If this is the case, LKO mice would reveal previously unappreciated aspects of the integumentary system [97,116].

## Figures and Tables

**Table 1:** Distribution of major CE proteins in various human tissues.

Origin		Involucrin	Loricrin
Hair follicle	**Interfollicular**	**+**	**+**
	**Infundibulm**	**+**	**+**
	Sebaceous gland	+	−
	Inner root sheath	+	−
	Hair shaft	+	−
Oral/throat	**Hard plate**	**+**	**+**
	Buccal	+	−
	Phalynx	+	−
Upper digestive tract	Esophagus	+	−
	Stomach	−	−
	Duodenum	−	−
Lower digestive tract	Colon	−	−
	Anus	+	−
Genital	**Foreskin**	**+**	**+**
	Cervix	+	−
	Vagina	+	±
Eye	Conjunctiva	+	−
Lymphoid organ	Thyms (mTEC *)	+	−
	Lymph nodes	−	−
	Spleen	−	−
Solid organ	Liver	−	−
	Pancreas	−	−
	Kidney	−	−
Urothelium	Urinary bladder	+	−
Endocrine organ	Thyroid	−	−
	Adrenal gland	−	−

* Medullary thymic epithelial cells. Bolded values: Loricrin expression is present as per immunohistochemistry.

**Table 2:** Epidermal differentiation and mouse models.

Target		Primary Defect		Remarkable Feature	Reference PMID
		Permeability Barrier	CEs		
**Gene Knockout**					
Cornified cell envelope (CE) components	Envoplakin (EPL)	−	−	Proportionally increased immature CEs	11564887
	Periplakin (PPL)	−	−	N/A *^1^	15226441
	Involucrin (IVL)	−	−	N/A	11038184
	EPL/PPL/IVL	+	+	Atopy, Resistant against chemically induced cutaneous carcinogenesis	18166659 24843010
	Loricrin	−	+	Delayed acquisition of permeability barrier, Compensatory response, ultraviolet B susceptible	11038185 23237955 27167730 29932941
	Filaggrin	−	−	Ichthyotic phenotype	22409988
	Caspase 14	+	− *^2^	Ultraviolet B susceptible, decreased urocanic acids, and free amino acids	17515931 21654840
Transglutaminase (TG)	TG1	+	+	Neonatal lethality	9448282
	TG3	−	−	Impaired hair development	22496784
Lipid transport	ABCA12	+	−	Neonatal lethality	18957418 18802465 27551807
	TMEM79	−	N/A	Mast cell-mediated histamigenic itch	30463955
**Transgenic overexpression**					
CE components	Involucrin	N/A	N/A	Alopecia, scaly epidermis	8405770
	Mutant loricrin	+	N/A	Mimicking Vohwinkel syndrome	11038186
	Human loricrin	N/A	N/A	Normal phenotype	8248167
	Filaggrin	N/A	N/A	Enhanced TEWL *^3^ recovery	15304104

*^1^ Not applicable. *^3^ Transepidermal water loss. *^2^ Abnormal keratohyalin granule that resemble TG1-deficiency is present.

**Table 3:** KEAP1/NRF2 system.

Target	Susceptibility/Challenge	Remarkable Feature	Reference PMID
**Gene Knockout**			
Nuclear factor erythroid 2-related factor 2 (*Nrf2*)	Ultraviolet B	Impaired cytoprotection	18200051
	Chemically induced cutaneous carcinogenesis		11248092
	Topical imiquimod	Dysregulated innate immunity	31953037
	Contact hypersensitivity	Outcomes may depend on contexts.	18325578
Kelch-like erythroid cell-derived protein with cap´n´collar homology-associated protein 1 (*Keap1*)	N/A *	Hyperorthokeratosis with loricrin overexpression and postnatal lethality	14517554
**Transgenic Overexpression: Dominant-Negative NRF2**			
Basal keratinocytes (K14 promoter-driven)	Chemically induced cutaneous carcinogenesis	Wound healing is not affected	16648473
Stratum granulosum keratinocytes (Loricrin promoter-driven)	Epidermal barrier recovery	Loricrin-knockout background can lead to postnatal lethality	23237955
**Transgenic Overexpression: Constitutively Active NRF2**			
Basal keratinocytes (K5 (or K10) promoter-driven)	ultraviolet B (resistant)	Phenotypes resemble autosomal recessive congenital ichthyosis or chloacne	20478997 22383093 24503019

* Not applicable.

**Table 4:** Vitamin A (retinoic acid) signaling pathway.

Target/Gene Targeting Strategy	Remarkable Feature	Reference PMID
Retinoic acid receptor α (RARα) (Dominant-negative, K1 promoter driven)	Defective permeability barrier and premature cornification that resembles vitamin A deficiency	7867929
9-*cis* retinoic acid receptor α (RXRα) (K10 promoter driven)	Attenuated response to topical all-*trans* retinoic acid (ATRA)	9000050
RXRα-conditional knockout (Tamoxifen-inducible K14-Cre-driven)	Vitamin D receptor-dependent atopic dermatitis	16199515

## Abbreviations

SC	stratum corneum
CE	cell envelopes
LG	lamellar granule
SG	stratum granulosum
ARCI	autosomal recessive congenital ichthyosis
LOF	loss-of-function
*ABCA12*	ATP binding cassette subfamily A member 12
*TGM1*	transglutaminase 1
LI	lamellar ichthyosis
IVL	involucrin
LOR	loricrin
EDC	epidermal differentiation complex
KIF	keratin intermediate filaments
mTECs	medullary thymic epithelial cells
FLG	filaggrin
KG	keratohyalin granules
SPRRs	small proline-rich proteins
VS	Vohwinkel syndrome
NLS	nuclear localization signal
LKO	LOR-knockout
LCE	late cornified cell envelope
UVB	ultraviolet B
KEAP1	Kelch-like erythroid cell-derived protein with cap´n´collar homology-associated protein 1
NRF2	NF-E2-related factor 2
ARE	antioxidant responsive element
EpRE	electrophile responsive element
LC	Langerhans cell
AD	atopic dermatitis
ACD	allergic contact dermatitis
*CASP14*	caspase-14
VAD	vitamin A deficiency
VDR	vitamin D receptor
RXRs	retinoid X receptors
PMNs	polymorphonuclear neutrophils
